# Single-stage total thoracoabdominal endovascular repair for acute non-A non-B aortic dissection with retrograde extension to the ascending aorta and concomitant abdominal dissecting aneurysm: a case report

**DOI:** 10.1186/s13019-026-04553-4

**Published:** 2026-07-14

**Authors:** Xiaojun Shu, Zuyue Huang, Dan Zhou, Yonglong Wang, Weijian Chen, Zhengfei Li, Yingxin Tan

**Affiliations:** 1 Department of Vascular Surgery, General Surgery, The First Hospital of Lanzhou University, No.1, Donggangxi Road, Chengguan District, Lanzhou, 730000 Gansu China; 2 The First School of Clinical Medicine, Lanzhou University, Lanzhou, Gansu China; 3 Heart Center, The First Hospital of Lanzhou University, Lanzhou, Gansu China; 4 School of Clinical Medicine, Tsinghua University, No.1, Donggangxi Road, Chengguan District, Beijing, Lanzhou, 730000 Gansu China

**Keywords:** Acute non‑A non‑B aortic dissection, Endovascular reconstruction, Physician-modified endograft, Spinal cord ischemia, Visceral artery revascularization

## Abstract

**Background:**

Acute non‑A non‑B aortic dissection represents a complex and high-risk condition. Traditional treatment protocols mandate emergency open surgical repair for any dissection involving the ascending aorta (Stanford Type A or DeBakey Type I/II). With ongoing advancements in endovascular devices, less invasive options are emerging as viable alternatives for select high-risk patients with ascending aortic involvement, potentially offering advantages over total arch replacement.

**Case presentation:**

A 73-year-old male presented with one week of chest and back pain and was diagnosed with acute non‑A non‑B aortic dissection with the primary entry tear located in the descending aorta (Ishimaru zone 3) extending retrogradely to the ascending aorta. Computed tomography angiography and three-dimensional reconstruction revealed an acute non‑A non‑B aortic dissection with a primary entry tear located at the Ishimaru zone 3, extending retrograde to the root of the ascending aorta and antegrade to the bifurcation of the iliac arteries. The left renal artery originated entirely from the false lumen, and the abdominal dissecting aortic aneurysm had a maximum diameter of 61 mm.Because the patient had not undergone any aortic imaging before symptom onset, it is not possible to definitively determine whether this lesion represents a pre-existing aneurysm or a chronic dissecting aneurysm with secondary false lumen dilatation. Based on the continuous dissection flap extending from the thoracic aorta into the infrarenal segment and the presence of partial false lumen thrombosis, we tend to favor that the 61 mm dilatation represents false lumen aneurysmal degeneration following the acute dissection. Given that the aneurysmal lesions in the visceral segment met established criteria for intervention and that staged repair carried a risk of interval rupture, a single-stage total endovascular reconstruction was performed following thorough discussion and at the request of the patient’s family. The reconstruction extended from the level distal to the origin of the left common carotid artery to the iliac bifurcation. A physician-modified endograft was deployed, and a fenestration was precisely aligned with the origin of the left subclavian artery, through which a Viabahn^®^ covered stent was placed to revascularize the left subclavian artery. For the visceral segment, a second physician-modified fenestrated stent graft with four fenestrations corresponding to the celiac artery, superior mesenteric artery, and bilateral renal arteries was used, with each branch reconstructed using a Viabahn^®^ covered stent. The patient recovered smoothly after surgery, with no signs of paraplegia, and was discharged one week post-surgery. At 1-year follow-up, satisfactory aortic remodeling and branch vessel patency were observed.

**Conclusions:**

This case report describes a patient with acute non‑A non‑B aortic dissection complicated by an abdominal aortic aneurysm who achieved a favorable outcome following single-stage endovascular repair. The patient did not develop paraplegia postoperatively, and following occlusion of the proximal thoracic aortic entry tear, the ascending aortic intramural hematoma resolved and underwent favorable remodeling. This case suggests that single-stage endovascular repair may serve as an alternative treatment option for patients at high risk for open thoracic surgery.

## Background

Acute non‑A non‑B aortic dissection with a primary entry tear at the Ishimaru zone 3 and retrograde extension into the ascending aorta represents a complex and high-risk condition [1]. Conventional management mandates urgent open surgical repair for any dissection involving the ascending aorta (Stanford type A or DeBakey type I/II) [2]. However, the classification and optimal management of non-A non-B aortic dissection remain controversial. Current therapeutic approaches encompass open surgical repair, thoracic endovascular aortic repair (TEVAR), hybrid procedures, and conservative medical management [3]. With the continuous advancement of endovascular devices, simplified and less invasive endovascular approaches are emerging as viable alternatives for selected high-risk patients with ascending aortic involvement, potentially offering advantages over complex arch replacement strategies.

## Case presentation

**Fig. 1:**
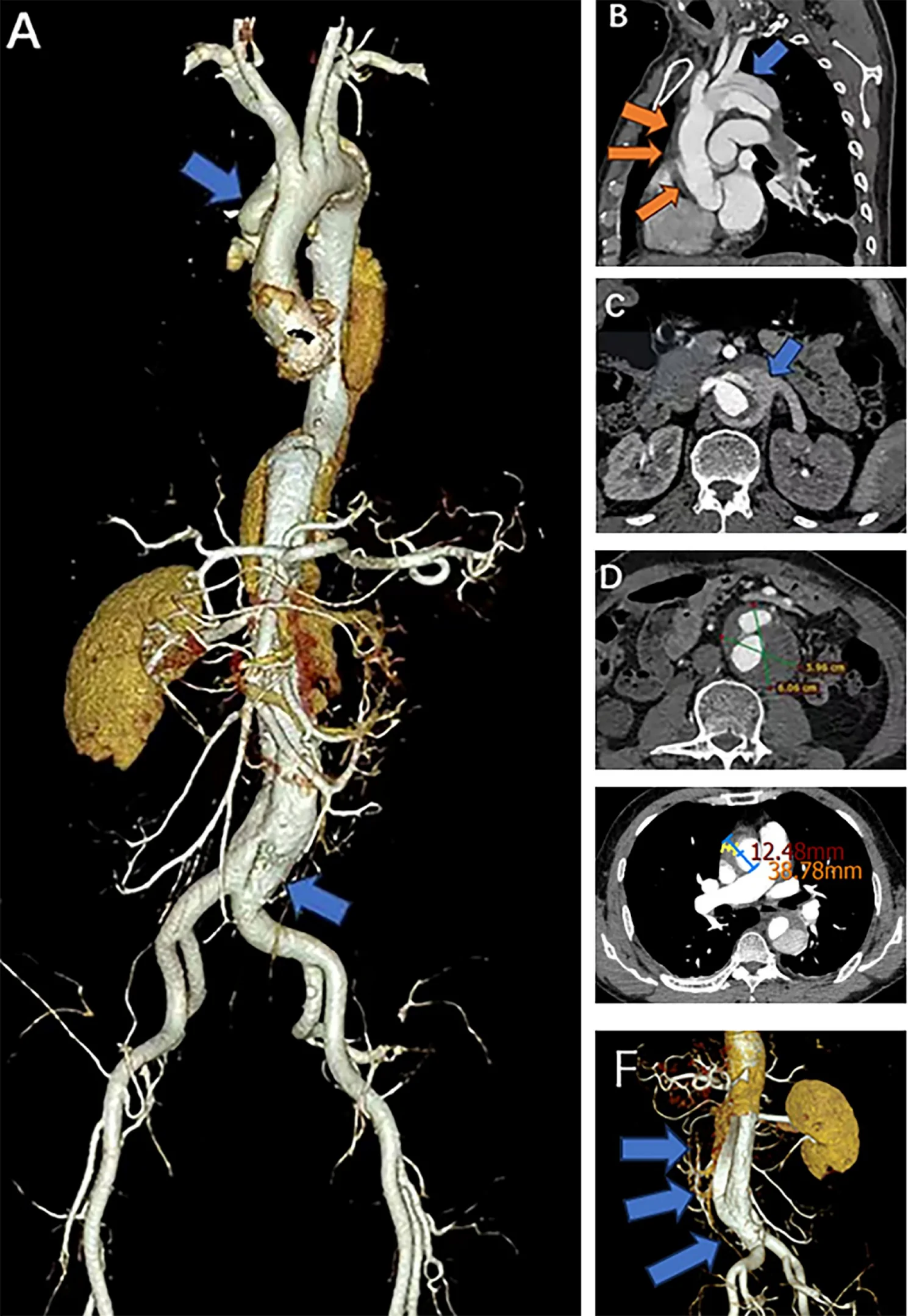
Preoperative computed tomography angiography and 3D reconstructions. (**A**) The 3D reconstruction shows an acute non‑A non‑B aortic dissection with the primary tear located in zone 3, extending retrograde to the aortic root and antegrade to the iliac bifurcation. (**B**) The coronal view shows that the dissection originates from the left subclavian artery. (**C**) Axial image showing that the left renal artery originates exclusively from the false lumen. (**D**) 3D reconstruction of a 61-mm abdominal aortic aneurysm. (**E**) Axial image of the ascending aorta showing a circumferential intramural hematoma. (**F**) Three‑dimensional reconstruction of the abdominal aorta showing the 61 mm dissecting aneurysm (arrow) extending from the infrarenal segment to the iliac bifurcation

A 73-year-old male presented with chest and back pain persisting for one week, corresponding to the acute phase (symptom onset < 2 weeks). Computed tomography angiography (CTA) and three-dimensional (3D) reconstruction revealed an acute non‑A non‑B aortic dissection with a primary entry tear at the Ishimaru zone 3. The dissection extended retrograde to the root of the ascending aorta, with circumferential intramural hematoma visible throughout the ascending aorta; the antegrade dissection extended to the iliac bifurcation (Fig. 1A and B). The left renal artery originated entirely from the false lumen (Fig. 1C). The abdominal aortic aneurysm had a maximum diameter of 61 mm (Fig. 1D). Without pre‑morbid imaging, the nature of the 61 mm abdominal lesion—pre‑existing aneurysm versus false lumen degeneration after acute dissection—cannot be definitively determined. The continuous dissection flap and partial false lumen thrombosis favor the latter. Circumferential intramural hematoma was also visible within the ascending aorta (Fig. 1E).The maximum diameter of the ascending aorta was 38.488 mm, and the maximal thickness of the intramural hematoma was 12.48 mm.

Despite the immediate initiation of aggressive medical treatment with beta-blockers upon admission to control blood pressure, the patient continued to experience chest pain. Given the clinical presentation and the fact that the abdominal aortic aneurysm diameter exceeded the threshold for endovascular treatment, surgical repair was recommended. The abdominal aortic dissecting aneurysm measured 61 mm in diameter, and the persistence of symptoms suggested aortic wall instability. To eliminate the risk of intraoperative rupture, following thorough discussion and at the family’s request, a decision was made to perform a single-stage total endovascular reconstruction. The patient has signed a written informed consent form for the surgery and the publication of the case report.

Based on preoperative CT and intraoperative DSA measurements, an ex vivo fenestration was created on the thoracic aortic stent (TAA2822B200, Ankura™, Lifetech, Shenzhen, China), with a custom 8-mm opening positioned at the corresponding site of the left subclavian artery origin. The opening was reinforced with contrast-enhancing marker coil sutures to facilitate intraoperative alignment. The stent graft was constrained with a constrainer, then reloaded into the original delivery sheath, and the constrainer was released.

**Fig. 2:**
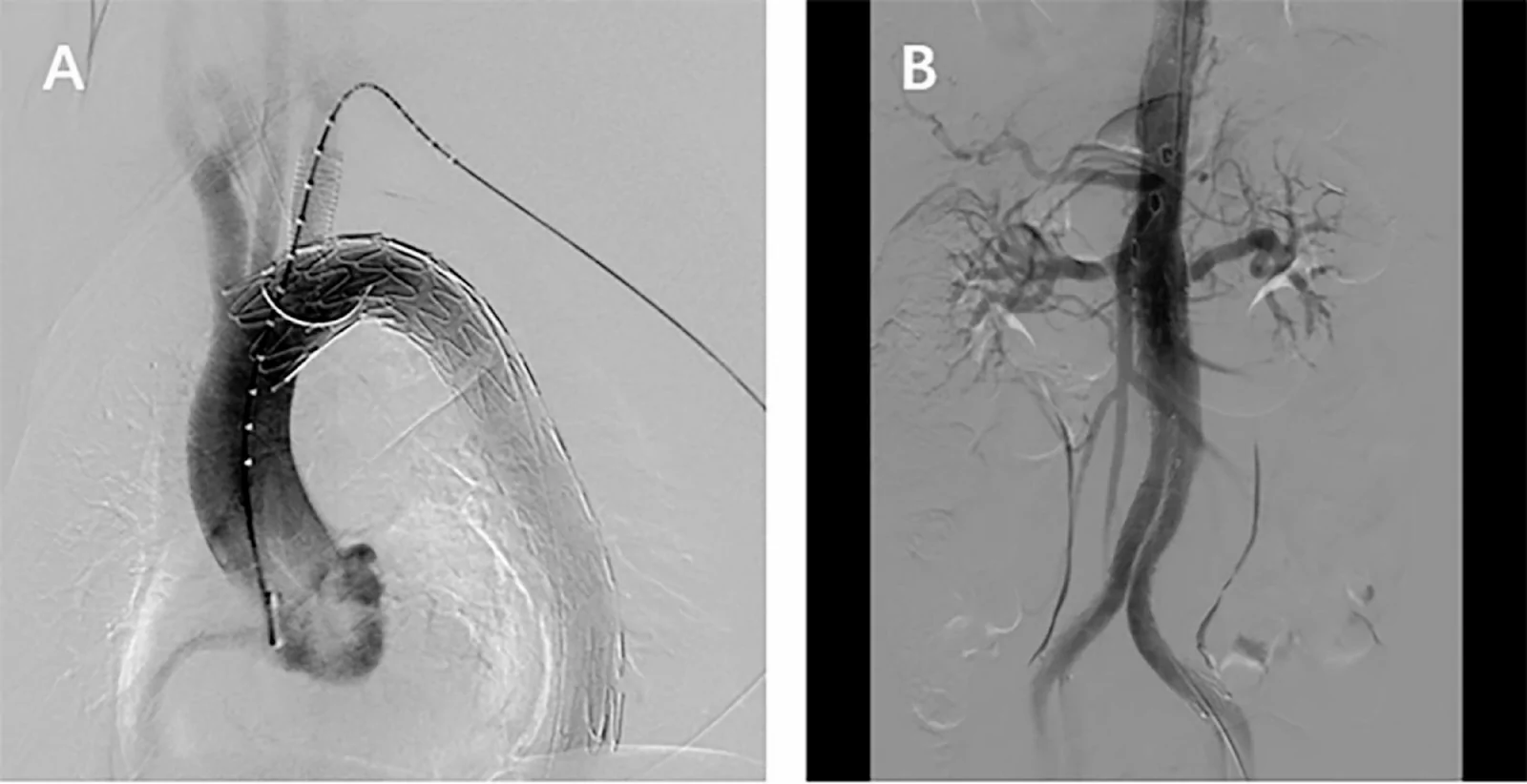
Single-stage total endovascular reconstruction. (**A**) Intraoperative angiography following deployment of the physician-modified thoracic endograft and left subclavian artery reconstruction. A covered stent (Viabahn^®^) was positioned through the fenestration to preserve LSA[Left subclavian artery] patency. (**B**) Completion angiography following visceral segment reconstruction. Four covered stents were deployed into the celiac artery, superior mesenteric artery, and bilateral renal arteries via corresponding fenestrations. A bridging covered stent graft was deployed to establish continuity between the arch and visceral stent grafts

Under general anesthesia, the physician-modified endovascular graft was deployed via the right common femoral artery. Under fluoroscopic guidance, precise alignment of the graft fenestration with the origin of the left subclavian artery was achieved. Via the left common femoral artery approach, a guidewire was advanced through the fenestration into the left subclavian artery with catheter assistance. A covered stent (Viabahn^®^, W. L. Gore & Associates, Flagstaff, Arizona, USA) was then deployed to reconstruct the vessel (Fig. 2A). Completion angiography confirmed successful occlusion of the proximal entry tear.

Based on preoperative CTA measurements, custom fenestrations were created on the second physician-modified endograft (Ankura™, Lifetech, Shenzhen, China) at the locations corresponding to the visceral arteries, and radiopaque marker coils were sutured to delineate the fenestrations. Precise fenestration alignment with the ostia of the celiac artery, superior mesenteric artery, and bilateral renal arteries was achieved under fluoroscopic guidance. Through left femoral access, guidewires with catheter support were selectively advanced into each branch via the corresponding fenestrations. To access the left renal artery, an 8 F steerable sheath (Fustar™, Lifetech, Shenzhen, China) was used with the stiff end of a 0.018-inch Command wire (Abbott Cardiovascular™, Lake County, IL, USA) to puncture the dissection flap to gain entry into the true lumen of the left renal artery. Four covered stents (Viabahn^®^, W. L. Gore & Associates, Flagstaff, Arizona, USA) were deployed into the visceral arteries (Fig. 2B). A covered stent graft (Ankura™, Lifetech, Shenzhen, China) was subsequently deployed to bridge the arch stent graft and the visceral stent grafts, thereby establishing continuity of the endovascular reconstruction.

Cerebrospinal fluid drainage was not performed for two reasons. First, revascularization of the left subclavian artery was achieved, which preserves antegrade vertebral and spinal perfusion. Consequently, the baseline risk of paraplegia was considered acceptably low. Second, the patient required immediate postoperative anticoagulation and antiplatelet therapy to prevent stent thrombosis and thromboembolic complications. Delaying these antithrombotic agents until after removal of a cerebrospinal fluid catheter (typically 48–72 h) would have exposed the patient to an unacceptable thrombotic risk. Moreover, combining cerebrospinal fluid drainage with early intensive antithrombotic therapy significantly increases the risk of spinal epidural hematoma. Therefore, we deliberately omitted cerebrospinal fluid drainage and implemented a multimodal spinal cord protection strategy consisting of permissive hypertension (mean arterial pressure > 90 mmHg), continuous intraoperative neuromonitoring, and left subclavian artery revascularization.

The total procedure duration was approximately 186 min. The fluoroscopy time was 31 min and 38 s, with a cumulative air kerma of 851.87 mGy and a cumulative dose-area product of 268.5 Gy·cm².

**Fig. 3:**
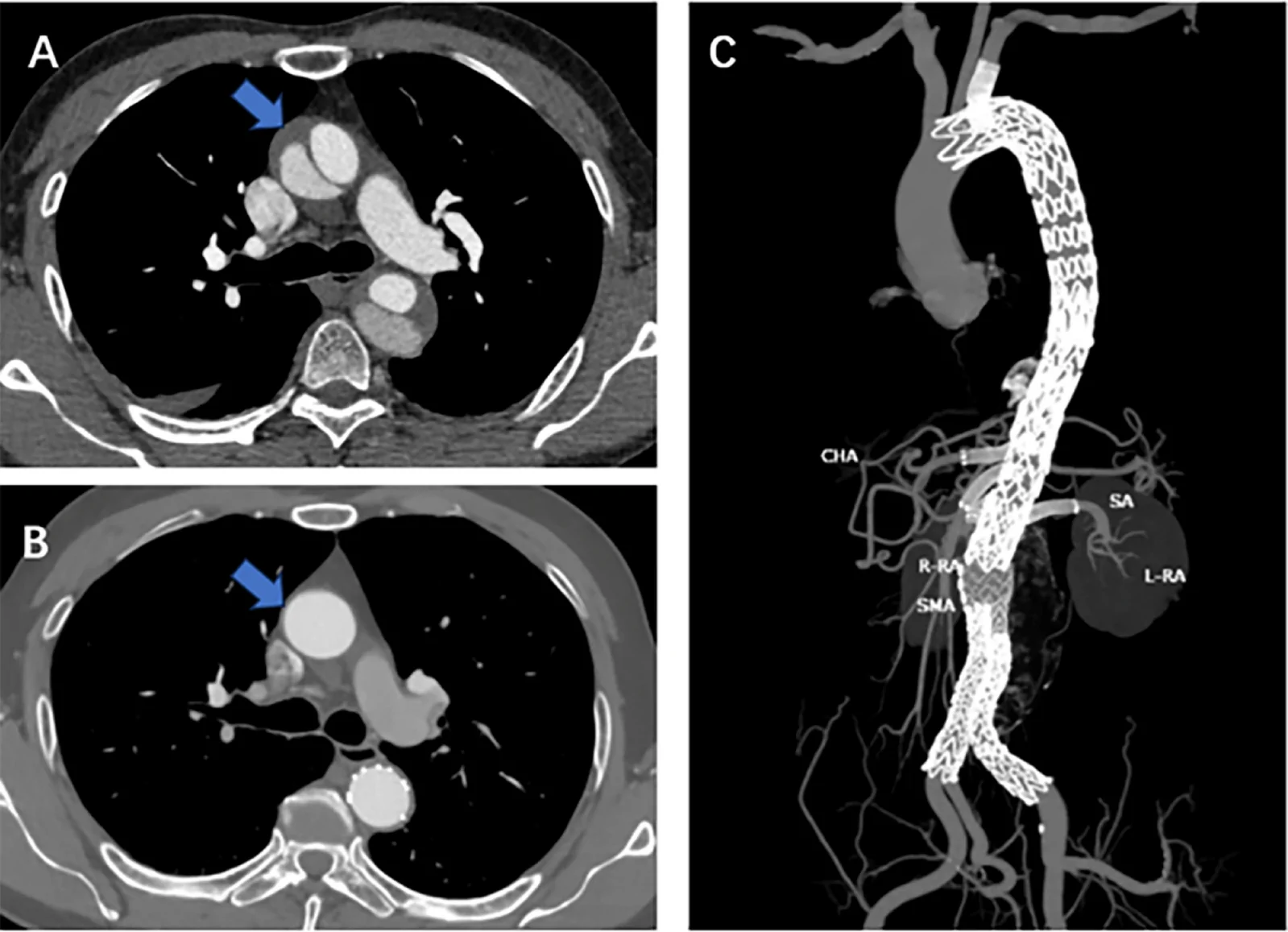
Postoperative follow-up computed tomography angiography. **A**, **B**) The 3-month postoperative follow-up shows good remodeling of the ascending aorta, with complete resolution of the intramural hematoma. **C**) The 1-year postoperative 3D reconstruction confirms that all reconstructed branches remain patent

Completion angiography following total aortic reconstruction confirmed complete false lumen exclusion with no evidence of internal leakage or endoleak. The patient experienced no complications such as paraplegia, hepatic insufficiency, or renal dysfunction and was discharged one week after surgery. At the 3-month follow-up, the ascending aorta demonstrated favorable remodeling (Fig. 3A and B), with complete resolution of the intramural hematoma. At the 1-year follow-up, all reconstructed vessels remained patent (Fig. 3C), and the patient reported no discomfort.

## Discussion and conclusions

The optimal management of non-A non-B aortic dissection with retrograde extension to the ascending aorta remains controversial, with endovascular repair emerging as a viable alternative in selected high-risk patients [3]. Single-stage total endovascular repair for this entity complicated by a concomitant abdominal dissecting aortic aneurysm is rarely reported in the literature. The present case illustrates the feasibility of endovascular management for this complex multisegment aortic pathology and highlights several strategic considerations.

The decision to perform single-stage total endovascular repair followed a structured clinical pathway. First, patient characteristics: this 73-year-old patient had hypertension, chronic kidney disease, and a circumferential intramural hematoma throughout the ascending aorta with no healthy proximal landing zone. Second, why open surgery was unsuitable: according to current guidelines [2], open arch replacement is the gold standard for any dissection involving the ascending aorta. However, the absence of healthy aortic tissue for anastomosis and the presence of extensive intramural hematoma rendered conventional arch replacement exceptionally high-risk, with prohibitive likelihood of anastomotic bleeding or iatrogenic dissection. Third, a staged approach was rejected because, according to STS/SVS guidelines, persistent pain despite medical therapy is a high-risk feature [4].; our patient’s symptoms did not subside with beta-blockers, indicating ongoing aortic wall instability. Moreover, staging would have exposed the patient to a 13% risk of interval aortic events (69% mortality if rupture occurred) [5]. and for a 61 mm dissecting aneurysm the annual rupture rate reaches 9–10% [6]. Delayed intervention was therefore deemed unsafe. Fourth, why single-stage total endovascular repair was chosen: after thorough multidisciplinary discussion, the patient’s family explicitly declined open surgery and accepted the risks of a single-stage procedure. Given our institutional experience with physician-modified endografts and the technical feasibility of total aortic reconstruction, we elected to proceed. This decision, while unconventional, eliminated the hazard of interval rupture and addressed both the proximal entry tear and the large abdominal aneurysm in one session.

Having established the rationale for urgent single‑stage repair, we now describe the technical challenges encountered and how they were addressed. Three specific technical challenges were encountered and successfully addressed. First, the left renal artery originated exclusively from the false lumen. Using an 8 F steerable sheath and the stiff end of a 0.018-inch wire, we performed a controlled puncture of the dissection flap under fluoroscopy, enabling placement of a covered stent from the true lumen into the left renal artery. Second, despite extensive aortic coverage, the patient did not develop paraplegia. Cerebrospinal fluid drainage was omitted due to the risk of epidural hematoma from postoperative antithrombotic therapy. Instead, a multimodal spinal cord protection strategy—permissive hypertension, left subclavian artery revascularization, and continuous neuromonitoring—was employed, aligning with Patel [7].

We selected physician-modified endografts (PMEGs) over commercial off-the-shelf devices or hybrid surgery because custom-made fenestrated/branched grafts require 6–12 weeks for manufacturing, rendering them unavailable for acute presentations. Off-the-shelf multibranched devices have restrictive anatomical criteria and do not accommodate a zone 2 landing zone with four visceral vessels. PMEGs were therefore the only viable endovascular option for this patient. The off-label nature of PMEG use was disclosed to and approved by our institutional ethics committee (No. LDYYLL2024-845). The patient provided written informed consent after detailed discussion of the investigational nature, potential risks (endoleak, misalignment, new entry tears, device integrity compromise), and the absence of approved alternatives. To minimize risks, we performed precise CTA-based fenestration planning, reinforced fenestrations with radiopaque markers, and deployed all grafts under slow, controlled fluoroscopy. Recent literature supports that PMEGs achieve outcomes comparable to factory-made fenestrated endografts in complex aortic repair, and they are a suitable option for emergent arch lesions [8]. Lifelong imaging surveillance is mandatory after PMEG implantation.

We did not directly treat the ascending aorta because this was a non-A non-B dissection: the primary entry tear was located in zone 3, and the ascending aorta presented only with an intramural hematoma (IMH), with no endothelial defect observed. The 2024 EACTS/STS guidelines recognize this condition and recommend TEVAR for complex cases accompanied by a descending aorta entry tear [2]. Multiple studies have demonstrated that TEVAR alone is sufficient. Koizumi et al. reported 19 patients treated with TEVAR, with no deaths and complete thrombosis of the ascending aortic false lumen at discharge [9]. Yang et al. found that TEVAR achieved complete resolution of IMH, with a significantly lower 30-day complication rate compared to open repair (7.7% vs. 39.4%, *p* = 0.037) [10]. Wang et al. observed positive signs of ascending aorta remodeling in 92.3% of 52 patients [11]. In this case, intraoperative DSA confirmed the absence of a tear at the ascending aortic entry or progression of IMH. CTAs at 3 months and 1 year postoperatively demonstrated complete resolution of IMH and good remodeling (Fig. 3A and B). Therefore, direct ascending aortic repair is neither necessary nor advisable, as it may cause harm.

Preoperative evaluation revealed an ascending aorta diameter of 38.78 mm, with no severe atherosclerosis or calcification, and an intramural hematoma thickness of 12.48 mm, indicating a moderate baseline risk. The primary entry tear was located at the root of the left subclavian artery. To ensure an absolutely safe landing zone, the stent would need to be placed at the 0-zone; however, this would require revascularization of the innominate artery and the left common carotid artery, thereby significantly increasing the complexity of the procedure. Therefore, we selected the distal left common carotid artery to cover the entry tear. To minimize the risk of RTAD, we employed a 0% oversizing at the proximal landing zone. A study by Xiang et al. demonstrated that oversizing > 5% was associated with a significantly higher cumulative incidence of RTAD (6.9% vs. 0.7%, *p* = 0.007) within one year after TEVAR for uncomplicated type B aortic dissection [12]. Following these preventive measures, no RTAD occurred in this patient.

Although this complex procedure required prolonged general anesthesia, the single-stage repair strategy minimized the patient’s cumulative risks, including repeated general anesthesia and multiple vascular access procedures inherent in a staged approach.

## Data Availability

The datasets used and/or analyzed during the current study are available from the corresponding author on reasonable request.

## Abbreviations

3D: Three-dimensional
CTA: Computed tomography angiography
LSA: Left subclavian artery. PMEG: Physician‑modified endograft
IMH: Intramural hematoma
RTAD: Retrograde type A dissection
